# Cluster sets vs. traditional sets: Levelling out the playing field using a power-based threshold

**DOI:** 10.1371/journal.pone.0208035

**Published:** 2018-11-26

**Authors:** James J. Tufano, Matej Halaj, Tomas Kampmiller, Adrian Novosad, Gabriel Buzgo

**Affiliations:** 1 Department of Physiology and Biochemistry, Faculty of Physical Education and Sport, Charles University, Prague, Czech Republic; 2 Department of Track and Field, Faculty of Physical Education and Sport, Comenius University, Bratislava, Slovak Republic; 3 Department of Sport Kinanthropology, Faculty of Physical Education and Sport, Comenius University, Bratislava, Slovak Republic; University of Guilan, ISLAMIC REPUBLIC OF IRAN

## Abstract

Cluster sets allow for velocity and power output maintenance, but the literature routinely uses highly fatiguing traditional set protocols. Although such studies have merit, others suggest fatigue should be avoided when training to improve power output, making those cluster set studies less practical. Therefore, the purpose of this study was to compare these set structures when truncating sets using a power-based threshold. Nine males (23.4 ± 0.6 yr) with various sport backgrounds performed 6 sets of back squats with individualized loads that elicited the greatest mean power (MPmax) output (112.7 ± 12.1% of body mass). Each set during the traditional set (TS) protocol included as many repetitions as possible until two consecutive repetitions dropped below 90% MPmax, which was followed by 120 s inter-set rest. The design was identical for cluster sets (CS) but with an additional 20 s intra-set rest after every 2 repetitions. The number of repetitions performed, mean velocity, and mean power output, were analyzed using 2(protocol)*6(set) repeated measures ANOVA. The number of repetitions during CS (51.8 ± 14.4) was greater than TS (31.9 ± 3.7) (*p* = 0.001), but the average velocity (CS = 0.711 ± 0.069, TS = 0.716 ± 0.081 m·s^-1^; *p* = 0.732) and power output (CS = 630.3 ± 59.8, TS = 636.0 ± 84.3 W; *p* = 0.629) of those repetitions were similar. These data indicate that CS are a viable option for increasing training volume during contemporary training where sets are ended when repetitions drop below velocity or power thresholds.

## Introduction

As an athlete’s ability to produce force quickly is important during many athletic movements, strength and conditioning professionals implement various exercises during training that should ultimately enhance the power-generating abilities of muscles during competition. For example, it has been suggested that resistance training should include a variety of exercises spanning across the entire force-velocity spectrum [1, 2], meaning that a well-rounded program could include exercises ranging from heavy squats and deadlifts to moderately heavy Olympic weightlifting derivatives to bodyweight jump squats and even assisted movements [2–4]. Regardless of the exercise or the external load, performing multiple repetitions with maximal concentric effort results in fatigue and a concurrent decrease in movement velocity and power output [5–7]. To ameliorate fatigue and combat these acute decreases in performance, the use of cluster sets has become increasingly popular within the strength and conditioning literature and within training environments [6].

Contrary to traditional sets where repetitions within a set are performed consecutively, a long inter-set rest period is provided, and another set of repetitions is performed consecutively, cluster sets include short, intra-set rest intervals, which likely allow for immediate energy stores and subsequent performance to be better maintained [6, 8, 9]. Likely as a result of more constant energy stores within the active muscle, recent research has identified that intra-set rest intervals can allow for greater loads for a given number of repetitions [7] or a greater number of repetitions for a given load [10]. Although these findings may play a role in developing strength, hypertrophy, or both, cluster sets are often implemented during power-focused training [6], where repeated exposure to maximal-velocity movements against a given load is desired. In support of this, the cluster set literature has repeatedly shown that movement velocity and power output are better maintained when utilizing intra-set rest or having more frequent inter-set rest periods [11–14]. Although adding intra-set rest intervals is easy to implement (i.e. only needing a mental countdown or, at most, a stopwatch), coaches who prefer to utilize technology during training may wish for a more objective approach for monitoring or guiding training. Therefore, this valid concern could mean that the addition of intra-set rest intervals using cluster set methods may reduce acute training stressors and fatigue so much that athletes may unwittingly elude an overload stimulus, resulting suboptimal adaptations. Therefore, to counteract any inadvertent and extreme over- or under-loaded stimuli, coaches may wish to monitor movement velocity or power-output to allow for training to be adjusted using objective data.

The recent surge of velocity-based training (VBT) in the literature and in practice serves as evidence to support the desire of coaches to objectively assess an athlete’s performance during training sessions [15–20]. Among these studies, velocity- or power-based thresholds are often used to truncate an exercise once a certain amount of fatigue has ensued, something that has not been implemented during traditional sets in cluster-set-focused research. Although VBT studies make use of recent technological advancements, some coaches may err on the side of caution and may not implement VBT due to its heavy reliance on technology and the fact that technology can fail unexpectedly. In these cases, it is possible that cluster sets could be used as an “a-priori” alternative to VBT, as a recent study showed that 12-second inter-repetition rest periods allowed for 36 consecutive back squat repetitions to be performed with 75% 1RM without dropping below a 20% velocity-decrease threshold [14], which has been suggested by previous VBT researchers [21, 22]. When the same study implemented 52.5 s of rest between 9 sets of 4 repetitions, only 28 of the 36 repetitions were performed above the 20% velocity-decrease threshold, indicating that more frequent rest periods are beneficial for maintaining movement velocity when the total rest time and number of repetitions are equal. Paradoxically, that study [14] as well as many others have utilized either the same loads for all subjects [12, 23] or loads relative to a subject’s 1 repetition maximum (1RM) [5, 13, 24], but aim to investigate the effects of cluster sets on maximizing power-output, with no studies utilizing loads that maximize power output [6, 11, 25, 26]. As power output varies between exercises and individuals, it would be logical to utilize loads at which an individual’s maximum power output occurs when determining the effects of cluster sets on power output. Additionally, it may be useful to combine the ideas of cluster set training and VBT to investigate the effect of cluster set structures not only in repetitions to failure or across a prescribed number of repetitions [6], but during a training session that employs a power-based threshold [21] where the traditional set structure also utilizes the threshold, possibly “levelling out the playing field”.

Therefore, the purpose of this study was to investigate the effects of cluster sets and traditional sets on velocity, power output, and training volume when using individualized loads at which mean power output is maximized. Also unique to this study, the protocols were based on a VBT approach whereby a decrease in mean power output below a certain threshold truncated each set, meaning that the number of total sets was prescribed, but the number of total repetitions was not. Based on previous research [10], we hypothesized that cluster sets would allow for greater movement velocities, greater power outputs, and greater total training volume compared to traditional sets, even when both protocols adopt the same power-threshold approach.

## Materials and methods

To investigate the effects of set structure on velocity, power output, and training volume when using power-based thresholds, this study employed a repeated measures research design. First, subjects completed a familiarization session where each subject performed back squats with progressively increasing loads to determine the individualized load at which mean power output was the greatest (MPmax). This load was then used during the traditional set (TS) and cluster set (CS) protocols, which were performed in a counter-balanced order and occurred approximately 72 hours apart.

**Fig 1:**
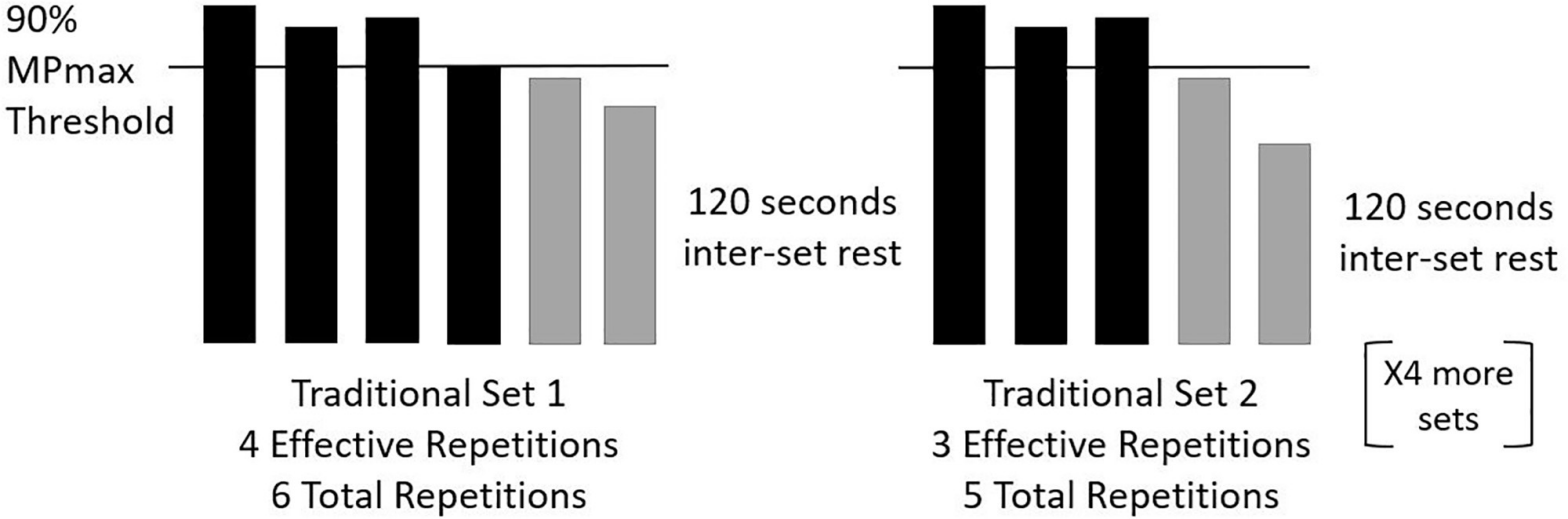
Example of the traditional set (TS) protocol with a threshold set at 90% of an individual’s maximal mean power output (MPmax). Each set was truncated when two consecutive repetitions dropped below 90% MPmax. The y-axis is theoretical mean velocity and each bar represents an individual repetition.

During the TS protocol, subjects performed 6 sets of back squats with their individualized MPmax load. Subjects completed each set with as many repetitions as possible until mean power output dropped below 90% of MPmax for two consecutive repetitions, as previous researchers have recommended that when developing “speed-strength” abilities, resistance training should be adjusted to maintain at least 90% of maximal mean power output [27–30]. After two consecutive repetitions were performed below 90% of MPmax, the set was concluded, and 2 min of inter-set rest was provided. This procedure was repeated for the remaining 5 sets (Fig 1).

**Fig 2:**
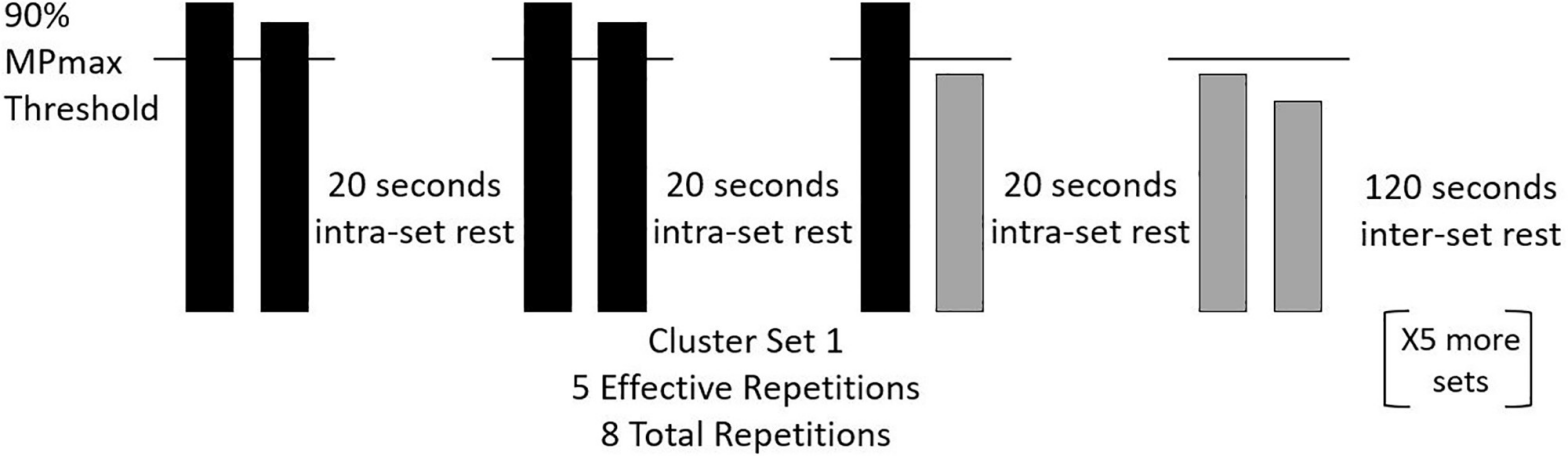
Example of the cluster set (CS) protocol with a threshold set at 90% of an individual’s maximal mean power output (MPmax). Each set was truncated when two consecutive repetitions within the same cluster dropped below 90% MPmax. The y-axis is theoretical mean velocity and each bar represents an individual repetition.

During the CS protocol, an undetermined number of clusters of 2 repetitions were performed with 20 s intra-set rest until both repetitions in each cluster were performed below 90% of MPmax. When this happened, the set was concluded, and 2 min of inter-set rest was provided. This procedure was repeated for the remaining 5 sets (Fig 2).

### Subjects

Ten university-aged males (23.4 ± 0.6 yr, 182.8 ± 2.7 cm, 79.39 ± 5.83 kg) with various specialized sport backgrounds (mainly track & field and soccer) participated in the study. All subjects routinely performed resistance training as part of their general training program for a minimum of at least 18 months prior to the commencement of this study, had no recent musculoskeletal injuries, and must have been able to perform a full barbell back squat with the hips descending below the knees with more than 100% of their body mass. The load with the greatest mean power output was 112.7 ± 12.1% of body mass. Subjects were instructed to refrain from any type of fatiguing lower body activity for the duration of the study, and all subjects read and signed an informed consent form that was approved by the Comenius University in Bratislava, Faculty of Physical Education and Sport ethics committee (project 4/2018).

### Measurements and procedures

#### Back squat exercise

Contrary to most cluster set studies where subjects were instructed to keep their feet flat on the floor to control the distance and technique of each squat [5, 13, 14, 31, 32], this study utilized a high-bar back squat with a calf raise. Considering the purpose of this study was to maximize power output, subjects were instructed to control the eccentric phase of the squat, and then to perform the concentric phase as explosively as possible, even forcefully plantar-flexing the ankles so that acceleration during the concentric phase was maximized. The average eccentric phase of each squat (i.e. depth of the squat) was 72.46 ± 5.90 cm, and the total displacement during the concentric phase was 84.69 ± 6.08 cm, meaning that the average displacement of the barbell after the completion of the squat (i.e. from the starting position until the highest point of the lift which occurred at the end of the calf raise) was approximately 12 cm. As this exercise included triple extension of the hips, knees, and ankles, the principle of training specificity indicates that the squats performed in this study were executed as a “speed-strength” exercise compared to a standard back squat that doesn’t involve ankle plantar flexion.

#### Warm-up

Before all sessions, subjects performed a 5 min general warm-up, followed by 5 bodyweight lunges on both legs, 5 bodyweight squats with a calf-raise, and 5 bodyweight jump squats with maximal effort in the concentric phase. Next, in the TS and CS sessions, subjects performed 2 repetitions with a barbell (20 kg) followed by 2 repetitions with 50%, 75%, and 100% of their individualized MPmax load, which was determined during a familiarization session described below. Between each warm-up set, 1 min of rest was provided, and every warm-up repetition was performed with maximal concentric effort.

#### Session 1: Familiarization and diagnostic session

This diagnostic session was performed to determine each subject’s MPmax load and included a progressive loading test whereby the barbell load increased by 10 kg increments, starting with a 20kg barbell [33]. After completing the general (e.g. 15 minutes that included jogging and dynamic stretches of the lower limbs) and exercise-specific warm-up (e.g. bodyweight lunges, calf raises, squats, and jump squats) subjects un-racked the bar, stepped backwards onto a standardized line to ensure similar foot placement, and started the repetition on a verbal signal from the researcher. At each load, subjects performed a single repetition with the eccentric phase under control and maximal concentric effort finishing with plantar flexion without jumping. A linear position transducer (details below) was used to provide instantaneous computations of mean velocity and mean power output. This process continued with 90 s of rest between each repetition until a subject’s individual load-power graph displayed a decrease in power output at two consecutive 10 kg increments. On average, subjects reached their MPmax of 763.2 ± 77.9 W with 89.5 ± 11.7 kg with a mean velocity of 0.86 ± 0.06 m·s^-1^, values that are somewhat lower than previous studies, but can likely be explained by the training status of our subjects (track and field athletes and soccer players) compared to previous studies that used resistance-trained men [24, 32]. Although we did not assess maximal strength (i.e. 1RM) in this study, it is possible that the MPmax loads were below the 70% or 75% 1RM loads that were used in previous studies. Additionally, as this was the first study to investigate the effects of back squats with a calf raise, the presence of plantar flexion, intent of plantar flexion, or both may influence strength, movement velocity, or power output compared to traditional back squats that are often used in research.

#### Traditional set and cluster set sessions

As explained above, the TS protocol consisted of 6 sets with 2 min inter-set rest intervals, stopping each set when MPmax dropped below 90% for two consecutive repetitions. The CS protocol was identical to TS, but repetitions were performed two at a time with 20 s of intra-set rest. During each protocol, subjects casually walked around the laboratory during their 2 min inter-set rest periods until about 10 s remained, at which point they began to return under the bar. During the CS protocol, subjects also casually walked around the laboratory during their 20 s intra-set rest periods. When there was 7 s left (in both the intra- and inter-set rest periods), subjects un-racked the bar, took one step backwards, and waited to perform the first repetition when the researcher’s countdown reached “0”, upon which the subject immediately started to perform the eccentric phase of the first repetition. As repetitions were performed consecutively, there was approximately a 1 s pause between each repetition to allow the subjects to reset themselves before the next repetitions and to allow the transducer to recognize the completion of one repetition and the beginning of the next. As soon as the bar was re-racked, the appropriate intra- or inter-set timer started. Like during the diagnostic session where all of the subjects were familiarized with the protocols and procedures, the start of each repetition of every set was verbally signaled by the researcher.

#### Data acquisition

All data were collected using a FiTROdyne Premium linear position transducer (FiTRONiC, Bratislava, Slovakia), which is a reliably method for measuring velocity and power output [34]. Time and vertical velocity were directly measured, and power output was calculated as the product of force (barbell load) and velocity. Total work was measured using force (barbell load) and distance of the entire range of motion, including the calf raise. Immediate feedback was provided to the researchers after every repetition and subjects were informed whether the previous repetition was above or below 90% of their MPmax threshold. Verbal encouragement was provided throughout the protocols, but neither visual nor any other forms of feedback were provided to the subjects. After each protocol, the number of effective repetitions (i.e. above the 90% threshold), ineffective repetitions (i.e. below the 90% threshold), and total number of repetitions were recorded.

#### Statistical analyses

When analyzing the number of total repetitions performed, one subject was an outlier and performed over two standard deviations more than the average. Therefore, this subject was excluded from all analyses and data from the other 9 subjects were analyzed. Descriptive statistics were calculated for mean velocity (MV), mean power output (MP), eccentric depth (ECC), total work per repetition (TW), number of effective repetitions (NER), and number of total repetitions (NTR). Individual 2(protocol)x6(set) repeated measures ANOVA were used to evaluate MV, MP, ECC, TW, NER, and NTR, with an LSD post-hoc test when necessary. The alpha level was set at *p* ≤ 0.05 and all statistical analyses were performed using SPSS 22.0 (IBM, Armonk, NY). Effect sizes were calculated using Cohen’s *d* and can be interpreted as small (0.20–0.49), moderate (0.50–0.79), and large (≥0.80). A post-hoc power analysis using G*Power (3.1.9, Dusseldorf, Germany) revealed a power of 0.99 using the number of total repetitions as the main variable of interest, an alpha level of 0.05, and an f-value of 0.945 [35].

## Results

The NER during CS (30.1 ± 11.7 repetitions) was greater (*p* = 0.009, *d* = 1.27) than TS (19.1 ± 3.7 repetitions), but the MV (*p* = 0.317) and MP (*p* = 0.276) of the NEF were similar. However, TW and ECC of NER were greater (*p* = 0.025 and *p* = 0.017, respectively) in TS than CS. Means, standard deviations, and effect sizes are shown in Table 1.

**Table 1:** Effect sizes (*d*) for all variables during the traditional set (TS) and cluster set protocols (CS). Effective repetitions include all repetitions performed over 90% MPmax, and total repetitions include effective repetitions and all repetitions performed below 90% MPmax.

	Number of Effective Repetitions	Number of Total Repetitions
Mean Velocity (m·s^-1^)	CS: 0.751 ± 0.073	CS: 0.711 ± 0.069
	TS: 0.763 ± 0.082	TS: 0.716 ± 0.081
	*d* = 0.15 in favor of TS	*d* = 0.07 in favor of TS
Mean Power (W)	CS: 664.5 ± 57.0	CS: 630.3 ± 59.8
	TS: 677.1 ± 79.4	TS: 636.0 ± 84.3
	*d* = 0.18 in favor of TS	*d* = 0.08 in favor of TS
Total Work (J)	CS: 741.11 ± 74.77	CS: 737.73 ± 75.45
	TS: 752.21 ± 78.97*	TS: 751.32 ± 80.05**
	*d* = 0.14 in favor of TS	*d* = 0.17 in favor of TS
Eccentric Depth (cm)	CS: 72.07 ± 6.42	CS: 71.97 ± 6.44
	TS: 73.02 ± 6.01*	TS: 72.98 ± 6.05*
	*d* = 0.15 in favor of TS	*d* = 0.16 in favor of TS

Symbols indicate a significant difference between protocols *p* < 0.05*, *p* < 0.01**.

The NTR performed during CS (51.8 ± 14.4 repetitions) was greater (*p* = 0.001, *d* = 1.89) than TS (31.9 ± 3.7 repetitions), but the MV (*p* = 0.732) and MP (*p* = 0.629) of the NTR were similar. However, TW and ECC of NTR were greater (*p* = 0.006 and *p* = 0.014, respectively) in TS than CS. Means, standard deviations, and effect sizes are shown in Table 1.

**Fig 3:**
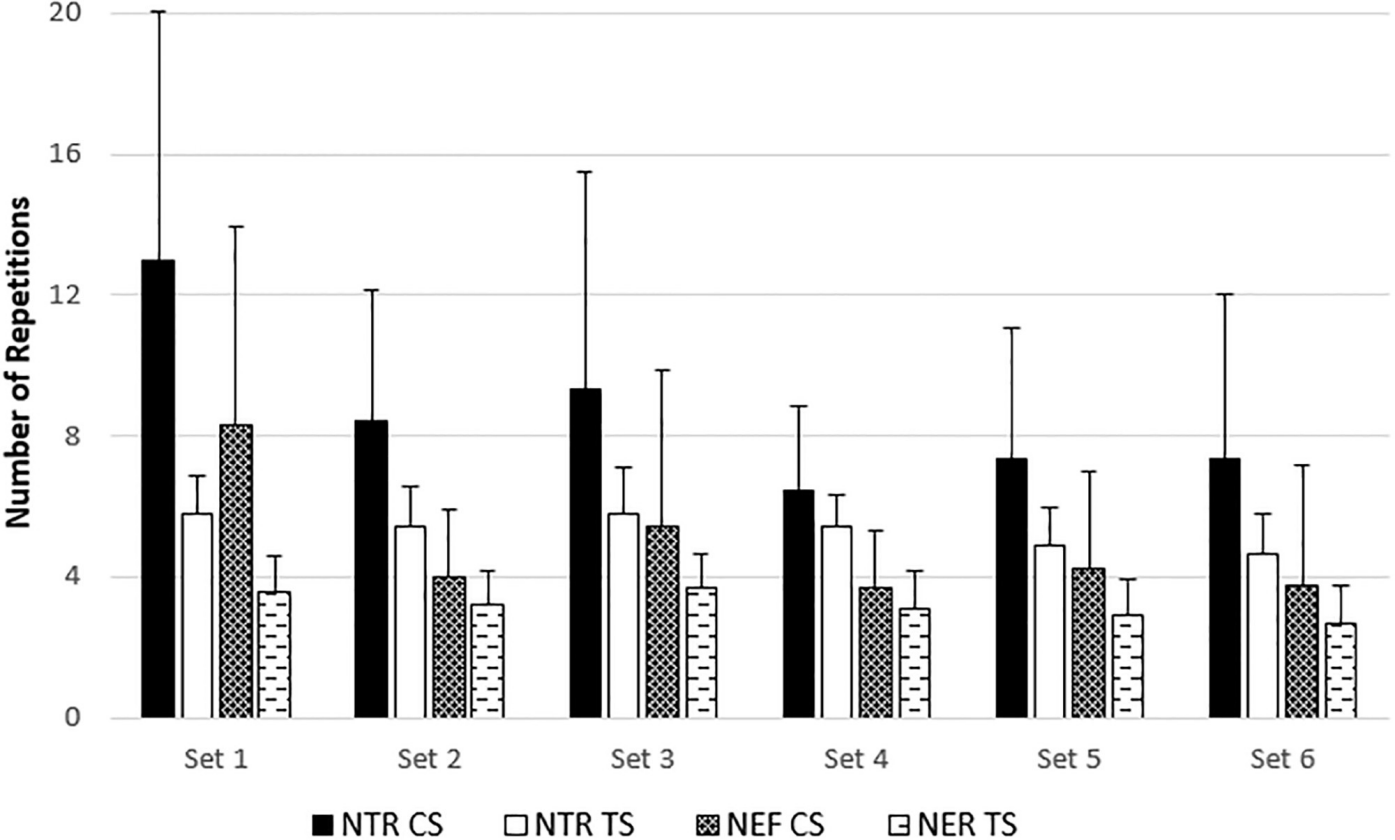
The number of total repetitions (NTR) and effective repetitions (NER) for the cluster set (CS) and traditional set (TS) protocols. Effective repetitions include all repetitions performed over 90% MPmax, and total repetitions include effective repetitions and all repetitions performed below 90% MPmax. Data are presented as mean ± standard deviation. There were no significant protocol*set interactions.

**Fig 4:**
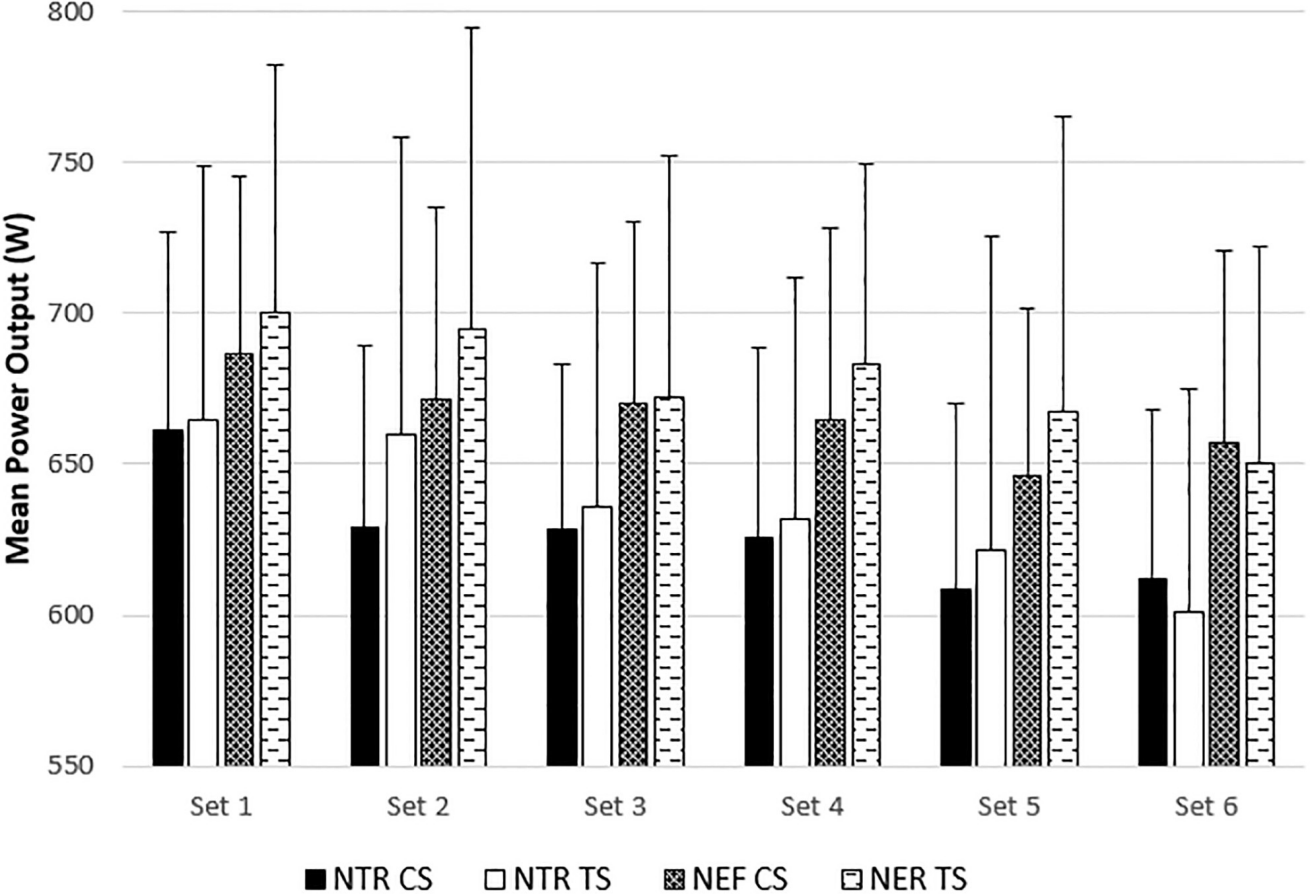
Mean power output for the number of total repetitions (NTR) and effective repetitions (NER) for the cluster set (CS) and traditional set (TS) protocols. Effective repetitions include all repetitions performed over 90% MPmax, and total repetitions include effective repetitions and all repetitions performed below 90% MPmax. Data are presented as mean ± standard deviation. There were no significant protocol*set interactions.

**Fig 5:**
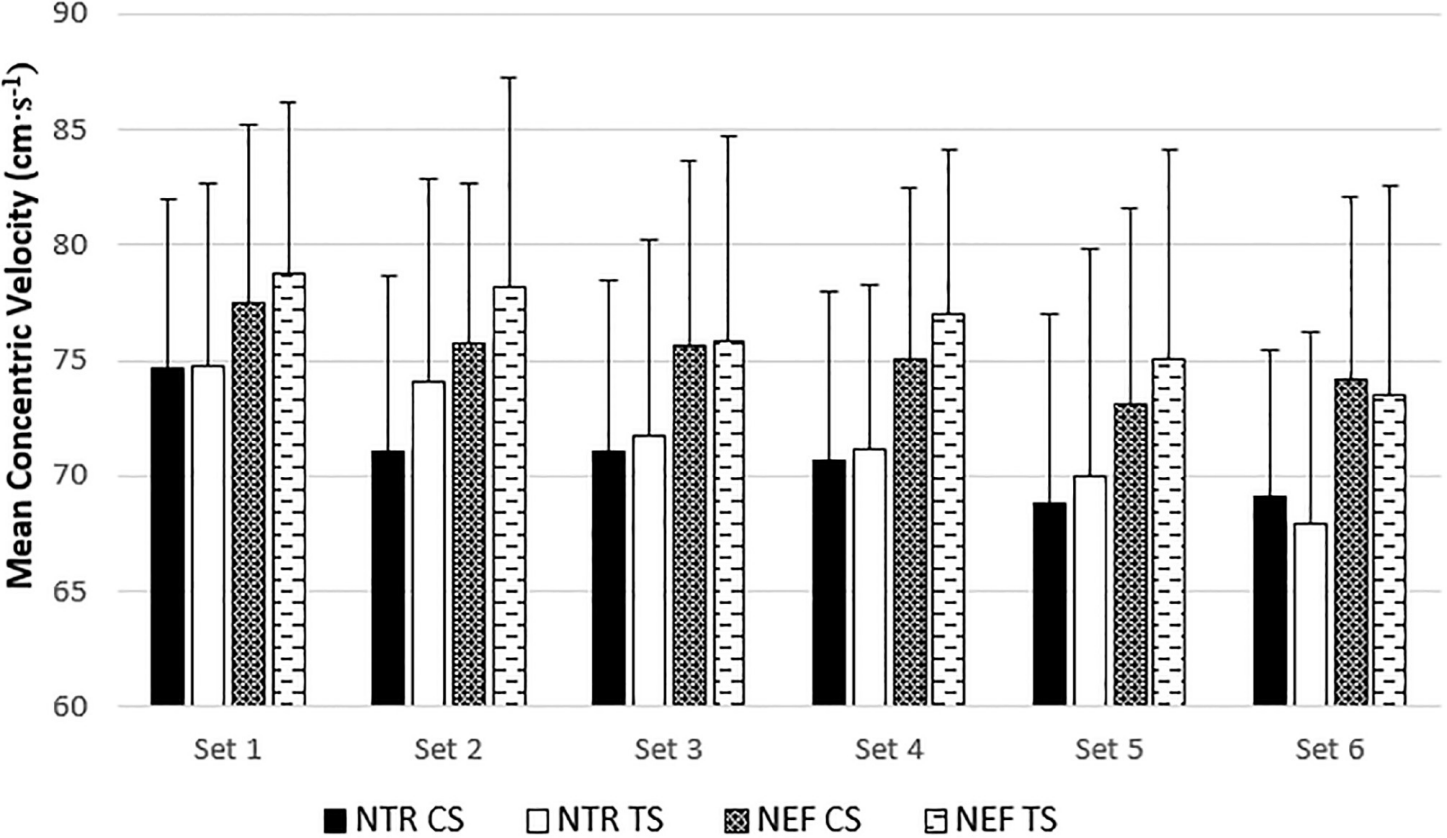
Mean concentric velocity for the number of total repetitions (NTR) and effective repetitions (NER) for the cluster set (CS) and traditional set (TS) protocols. Effective repetitions include all repetitions performed over 90% MPmax, and total repetitions include effective repetitions and all repetitions performed below 90% MPmax. Data are presented as mean ± standard deviation. There were no significant protocol*set interactions.

Set-by-set data for NTR and NEF, MV, and MP can be found in Figs 3, 4 and 5, respectively. Despite a greater NTR during CS in the first two sets (*p* = 0.011 and 0.027, respectively) and a greater NEF during CS in the first set (*p* = 0.027), these protocol*set interactions were not significant (*p* = 0.120 and 0.118, respectively). There were no interactions for MV or MP for either NTR or NER. The corresponding effect sizes are listed in Table 2.

**Table 2:** Set-by-set effect sizes (*d*) for all variables between the traditional set (TS) and cluster set protocols (CS). Effective repetitions include all repetitions performed over 90% MPmax, and total repetitions include effective repetitions and all repetitions performed below 90% MPmax.

		Number of Repetitions	Mean Power Output	Mean Velocity
Total Repetitions (CS—TS)	Set 1	1.42	0.04	0.02
	Set 2	1.09	0.37	0.37
	Set 3	0.80	0.11	0.09
	Set 4	0.55	0.09	0.07
	Set 5	0.88	0.15	0.12
	Set 6	0.78	0.16	0.16
Effective Repetitions (CS—TS)	Set 1	1.18	0.19	0.17
	Set 2	0.51	0.28	0.30
	Set 3	0.55	0.04	0.02
	Set 4	0.40	0.28	0.27
	Set 5	0.63	0.27	0.22
	Set 6	0.44	0.10	0.08

## Discussion

The current body of evidence overwhelmingly supports cluster sets over traditional sets when velocity and power maintenance are desired [6]. However, the large majority of these studies were designed so that the traditional set protocols were extremely fatiguing [5, 7, 13, 31]. This classical training approach has become challenged by research indicating that less-fatiguing resistance-training strategies impose similar, and at times superior, strength and power adaptations [36, 37]. Therefore, our study took a novel approach and implemented TS that were, by design, not extremely fatiguing. In doing so, our data show that when using a power-based threshold to truncate resistance-training sets, CS and TS resulted in similar movement velocities and power outputs by design. Interestingly, the effect sizes of the present study show a slight possible advantage (insignificant p-values and negligible-to-small effect sizes) for TS, which is in stark contrast to previous studies that often highlight the extreme fatigue that occurs in traditional sets. When repeatedly reading about the fatiguing-nature of traditional sets [6], readers ultimately believe that traditional sets are arduous and malevolent and that cluster sets are fatigue-resistant and steadfast. Although the present study indicates that CS and TS are in fact quite similar in terms of movement velocity and power output when using a power-based threshold, as expected, CS resulted in a significantly greater NER and NTR, indicating that total training volume was significantly greater during CS without decreasing acute repetition performance.

This study is not the first to show that more repetitions can be performed using cluster sets compared to traditional sets. Previous research that used cluster sets to redistribute rest periods and approximately equalize the work-to-rest ratio that occurred during traditional sets also showed that cluster sets enable more repetitions to be performed compared to traditional sets [10]. In that study, subjects performed 3 traditional sets of back squats to failure using a 4RM load with 3 min of inter-set rest. They later performed individual repetitions with the same work-to-rest ratio and number of repetitions as their traditional set protocol but were then allowed to continue performing individual repetitions with the same work-to-rest pattern until failure. As a result of having more frequent rest intervals, subjects were able to complete approximately 5 times the number of repetitions (45.0 ± 32.0) as they completed with traditional sets (9.3 ± 1.9) [10]. The present study shows that CS resulted in approximately 1.6 times more NER and NTR than TS, values that are comparatively dwarfed by the 5-fold increase noted in the aforementioned study. This discrepancy further illustrates the necessity of the present study, as using cluster sets to train to failure results in an anomalous number of repetitions compared to performing traditional sets to failure. By using velocity- or power-based thresholds in practice, cluster sets would likely not result in 5 times the number of repetitions as traditional sets, but rather a more modest but significant increase, possibly of about 1.6 times as seen in the present study. However, it is worth mentioning that the intra- and inter- subject variability for the number of repetitions performed during CS was quite large. For example, the NEF in the first CS set ranged between 2 and 21 repetitions, with the NTR ranging between 4 and 28, whereas the NEF in the first TS set ranged between 2 and 5 with the NTR ranging between 4 and 7. Specifically, one subject who completed 21 NER during the first CS completed 7 NER during the second set and only 2 NER during the final set. With this in mind, it is possible that the greater volume experienced during the first set of CS may have affected the performance of the subsequent CS in some subjects, despite having 2 min of inter-set rest. This notion is supported by the largest pro-TS effect sizes during the 2^nd^ set, when the accumulated fatigue of the 1^st^ CS set may have affected performance during the 2^nd^ CS set (Table 2). Therefore, strength and conditioning professionals should consider these individual differences and the possibility of accumulated fatigue when implementing similar protocols with their athletes.

Generally, performing more repetitions during cluster sets may seem intuitive, especially when the total rest time is greater. Moreover, previous research has shown that not only do cluster sets allow for more repetitions compared to traditional sets, but those additional repetitions are performed with greater movement velocities and presumably greater power outputs [10]. However, this this was not observed in the present study. In fact, although not significantly different, it is possible that the CS structure used in the present study may have had a slight negative effect on MV and MP compared to TS, demonstrated by effect sizes reaching up to 0.37 in NTR and up to 0.29 in NEF, both in favor of TS for MV and MP. As these differences were not significant, it would be inaccurate to claim that TS had greater MP and MV than CS. Nevertheless, it is possible that the greater NTR performed during CS may have resulted in slightly more accumulated fatigue throughout the session. Although ECC and TW per repetition were statistically greater during TS, a 1 cm change in squat depth is likely not practically significant, and likely does not indicate any more or less fatigue for either protocol. Therefore, it seems as though coaches must perform a balancing act between increasing training volume and maintaining power output, even when using power-based thresholds in traditional set and cluster set settings.

Unique to our study is the use of a MPmax load combined with a power-based threshold approach, two things that have not been investigated in the cluster set literature to date. Our data show that when using a power-based threshold to truncate traditional sets, cluster sets may not be as superior as many practitioners may have originally thought but may only be superior to traditional sets when traditional sets are designed to induce large amounts of fatigue. Although our study provides valuable insight and indicates that velocity- or power-based thresholds level out the playing field when comparing traditional and cluster sets, future studies should carefully consider their research design to sufficiently address the challenges at hand. For example, the decision to require two repetitions to fall below 90% of MPmax before ending a set in the present study was made to be confident that fatigue had in fact accumulated and would continue to build. However, this decision theoretically could have allowed for a single “bad repetition” that may have been performed at 89% MPmax followed by another repetition at 90% and so on. In doing so, it is possible to inadvertently hover around 90% MPmax despite performing each concentric phase at maximum effort, resulting in an increasing number of repetitions below the threshold, but not consecutively. Using such thresholds draws a fine and very murky line between what can be considered as sound scientific methodology (whereby crossing a threshold can be seen as black and white) and what can be done in practice (whereby a repetition performed at 89.4% MPmax is essentially the same as a repetition performed at 89.5% or 90% MPmax, for example). Therefore, future researchers should strongly consider this as the strength and conditioning field continues with threshold-based research. Another limitation of this study is the large inter-subject variability in the number of repetitions performed in the CS sets. As with most sport science research, individual data can be presented and analyzed, but coaches should take the next step by applying these novel training principles on an athlete-by-athlete basis, including other athletes from other sport backgrounds.

As this study compared TS and CS both using a power-based threshold approach, the next step researchers may wish to take is to investigate a TS protocol using a true VBT approach to a CS protocol that does not use a VBT approach, but instead either redistributes the total rest time to include shorter but more frequent sets [38] or includes additional intra-set rest periods [5], both of which have been shown to maintain velocity and power output. Conducting such a study would further elucidate whether specialized equipment is needed to objectively monitor fatigue and reactively truncate each set, or if proactively adjusting rest periods would be sufficient for maintaining velocity and power output for a given training volume. Additionally, as this study is the first to utilize individualized loads based on MPmax during cluster sets, our findings should be validated using other exercises and before the results from this study become well-accepted and implemented across a variety of exercises.

## Conclusions

This study indicates that when power-based thresholds are utilized, velocity and power output are equally maintained during cluster sets and traditional sets. However, cluster sets structures still allowed for a greater number of repetitions when using a 90% power-based threshold. Therefore, coaches and athletes can transfer these findings into practice by implementing cluster sets even during power- or velocity-based training when periods of greater training volumes are desired. However, when doing so, caution should be used as to not perform so many repetitions during cluster sets that they negatively affect the repetitions of subsequent sets.
